# Awareness and beliefs towards organ donation in chronic kidney disease patients in western India

**Published:** 2015-06-21

**Authors:** Manish R Balwani, Vivek B. Kute, Himanshu Patel, Pankaj R. Shah, Jitendra Goswami, Pravin Ghule, Maulin Shah, Vipul Gattani, Hargovind L. Trivedi

**Affiliations:** ^1^Department of Nephrology and Clinical Transplantation, Institute of Kidney Diseases and Research Center, Dr. HL Trivedi Institute of Transplantation Sciences (IKDRC-ITS), Ahmedabad, India

**Keywords:** Awareness, Chronic kidney disease, Organ donation policies

## Abstract

**Introduction:** There is a wide discrepancy between demand for and availability of donor organs for organ transplantation. There is no study on awareness about organ donation in chronic kidney disease (CKD) patients in India.

**Objectives:** To study the awareness and beliefs towards organ donation in CKD patients on hemodialysis in western India.

**Patients and Methods:** Authors conducted a cross sectional study among 85 CKD patients to evaluate knowledge about and attitude towards organ donation at a tertiary hospital.

**Results:** Age of respondents ranged from 15 to 75 years. All were aware of term organ donation and cadaver donation. About 47% of people heard about organ donation through hospital or from doctor. Strikingly, radio was not the source of information to any of the respondents, despite radio being one of the most common medium of mass communication. Almost one third of patients were unaware about any legislation regarding organ donation. All respondents felt that organs should go to the needy irrespective of their religion. About 70% feel that medical colleges should make decisions about organ donation in case of unclaimed dead bodies. About 31.76% believe that there is a danger that donated organs could be misused, abused or misappropriated.

**Conclusion:** Our study shows about 31.76% of our participants believe that there is a danger that donated organs could be misused, abused or misappropriated. There seems to be paucity of information and awareness regarding organ donation among CKD patients. Mass media, religious and political leaders may be involved to maximize awareness about organ donation.

**Implication for health policy/practice/research/medical education:**

As we expect that diseased population will have more knowledge about organ donation as compared to general public, but even affected population has less knowledge regarding legislation of organ donation. General public along with diseased population should be made aware of organ donation procedures and organ allocation policies with the help of medical, mass media and religious community involvement.

## Introduction

Organ transplantation is the preferred treatment modality for end-stage organ disease (1). It offers a better quality of life and has long-term survival benefits. Patients deemed fit for transplantation by the transplant committee often wait long for a donor organ. Inadequate supply of cadaver organs is especially vital for lung, heart and liver recipients, as these patients cannot be sustained for long on mechanical devices, unlike patients with chronic kidney disease (CKD) stage 5 who can be maintained on long-term dialysis. There is an increasing discrepancy between the available number of deceased donor organs and the number of patients on the waiting list for organ transplantation. Although the Indian population is well accustomed to the idea of blood donation, donation of solid organs after death continues to be in its infancy (2). There is a strong need for awareness about organ donation and its allocation policies among the general population in India along with CKD patients. One of the important factors that might contribute to this limited availability of donor organs is inadequate knowledge about the legal and procedural details of organ donation. An assessment of knowledge, attitudes and beliefs of CKD patients towards organ donation and its allocation would help to plan sensitization and increase awareness at the patient and family level and to propagate further knowledge at the community level. This is an cross-sectional study of organ donation with specific emphasis on the level of awareness, attitudes and beliefs among the CKD patients in one of the largest tertiary hospital and transplant center in western India.

## Objectives

To study the awareness and beliefs towards organ donation and its allocation in CKD patients in western India.

## Patients and Methods

A cross-sectional study was done among 85 CKD patients on hemodialysis in a tertiary care center in Ahmedabad city of Gujarat state of India. Ethical committee permission was obtained. Participants were randomly selected. All the selected participants gave their consent to participate in this study. Respondents were interviewed by the authors with a formulated questionnaire which was made keeping in mind the various local factors that can influence the awareness and belief about organ donation.

### Ethical issues

The research followed the tenets of the Declaration of Helsinki. Informed consent was obtained and the research was approved by the Ethics Committee of Institute of Kidney Diseases and Research Center, Dr. HL Trivedi Institute of Transplantation Sciences (IKDRC-ITS), Ahmedabad, India.

### Statistical analysis

Data entry was made in Microsoft Excel in codes and analysis was done by SPSS software.

## Results

Eighty-five stage 5 CKD (dialysis) patients were included in this study. Age of respondents participating in the study ranged from 15 to 75 years, maximum number of patients being in the age group of 30-45 years. Majority were males. About 82% belonged to Hindu religion. Around 60% of participants were married (Table 1).

**Table 1:** Socio-demographic characteristics of hemodialysis patients aware of the term organ donation

**Socio-demographic variable**	**Number (n=85)**	**Percent**
Sex		
Male	70	82.35
Female	15	17.65
Age		
15-30	7	8.23
30-45	33	38.82
45-60	24	28.23
60-75	21	24.70
Education		
Primary	19	22.35
Secondary	24	28.24
Higher education	9	10.59
Graduation	15	17.65
Post-graduation	12	14.11
Informal education	0	0
Diploma	0	0
Can read & write only name	0	0
Illiterate	6	7.05
Occupation		
Student	25	29.41
Housewife	9	10.59
Government of employee	6	7.06
Private employee	0	0
Volunteer	3	3.5
Self-employed	18	21.18
Retired	3	3.5
Unemployed	21	24.70
Religion		
Hindu	70	82.35
Muslim	15	17.65

### Awareness about organ donation

All respondents were aware of the terms ‘organ donation’ and ‘cadaver donation.’

### Source of information about organ donation

About 47% of people had heard about organ donation through a hospital or from a doctor. Audiovisual media such as television (21%), radio (0%) and newspaper/magazines (14%) were other sources of information. Around 14% of people had heard through a friend or a colleague (Table 2).

**Table 2:** Source of information about organ donation

**Sources**	**Number**	**Percent**
Heard from a doctor or in a hospital	40	47
Internet/online resources	3	3.52
TV	18	21.17
Radio	0	0.00
Newspaper/magazines	12	14.11
Friend or colleague	12	14.11

### Awareness about legislation

About 35.29% patients were unaware about any concerned legislation (Table 3).

**Table 3:** Are you aware of any local or international legislation with regards to organ donation?

**Variable**	**Number**	**Percent**
Local legislation	22	25.88
International legislation	3	3.52
Both from the above	30	35.29
None from the above	30	35.29

### Unclaimed dead bodies

About 67% participants were of view that in case of unclaimed dead bodies, decisions about organ donation should be done by medical colleges/hospitals/doctors whereas around 24% and 4% people said that it should be done by police departments and charitable organizations respectively (Table 4).

**Table 4:** Who should make decisions about organ donation in case of unclaimed dead bodies?

**Variable**	**Number**	**Percent**
Charitable organization	4	4.70
Medical colleges/government health institutions	57	67.05
Police	21	24.70
A judge	3	3.53
No one	0	0.00

### Fate of donated organ

About 32% respondents believed that there is a potential danger of donated organs being misused, abused or misappropriated (Table 5).

**Table 5:** Do you believe that there is a danger that donated organs could be misused, abused or misappropriated?

**Response**	**Number**	**Percent**
Never	55	64.70
Sometimes	27	31.76
Often	0	0.00
All of the time	3	3.52

### Religious factors

All respondents felt that the organs should go to the needy irrespective of their religion. About 78% respondents said that their religion allows for donation of organs which included both Hindus and Muslims (Table 6).

**Table 6:** Does your religion allow organ donation?

**Response**	**Number**	**Percent**
Yes	67	78.82
No	9	10.58
Don’t know	9	10.58

### Purpose of organ donation

About 92% participants said that organ donation is done to save some ones life (Table 7).

**Table 7:** Why is organ donation done?

**Variable**	**Number**	**Percent**
To save some one’s life	79	92.94
Out of compassion/sympathy	3	3.52
For money	3	3.52
As responsibility	0	0.00
Others	0	0.00

### Blood donation

No participant had history of blood donation when they were healthy.

### Organ donation registration

None of the respondents had registered for organ donation when they were healthy.

### Promotion of organ donation

One hundred percent of the participants were in the favor of promoting organ donation.

## Discussion

All our participants had heard about cadaveric organ donation. This can be attributed to their disease for which kidney transplant as a treatment option must have been explained to them by their treating physician. About 60% of respondents were aware of organ donation in general in a study done in Lagos, Nigeria (3). In our study, all participants felt that the cadaveric organs should go to needy recipients irrespective of their religion. This could be attributed to disease they are suffering from as they can well understand the importance of need of organ donation as compared to the general public. About 78% respondents said that their religion allows for organ donation. India is the world’s most culturally, linguistically and genetically diverse country. Religious beliefs and preaching have a greater impact on the attitude and behaviour of people. As per our knowledge, there is no religion which prohibits organ donation. This needs to be conveyed by religious and political leaders. All our respondents were in favor of organ donation and its promotion in the future. This is higher when compared to data from a study done in Brazil which reported that 87% of respondents were in favor of organ donation (4). In our study, 92.94% respondents said that organ donation is done for saving another human’s life whereas 3.5% replied that it is done for monetary gain. About 35.29% participants were unaware about national or international law regarding organ donation. Thus, spreading awareness regarding the act that laid down the guidelines for organ transplantation in India becomes quintessential in the study region to bust any myths or prejudices about organ allocation policies. In our study, 47% of people heard about organ donation through the hospital or a doctor. Audiovisual media such as television (21%) and newspaper/magazines (14%) were other sources of information about organ donation. Strikingly, radio was not the source of information to any of the respondents who participated in this study, despite radio being one of the most common medium of mass communication in India. About 14% of people heard about organ donation through a friend or colleague. In a study by Annadurai et al, 53% of the respondents heard about organ donation from print and electronic media while 34.1% heard from health care workers (5). About 67% said that medical colleges/government institutions should make decisions about organ donation in case of unclaimed dead bodies. About 32% believed that there is a danger that donated organs could be misused, abused or misappropriated. Therefore, it becomes essential to assure people that rich or influential persons are not given any priority; in reality the organ allocation system is blind to wealth or social status. Factors such as race, gender, and income or celebrity status are never considered when determining organ recipients. The organ allocation process needs to be transparent in order to gain confidence of the people. Regarding the practice of organ donation, none of our participants had practiced voluntary blood donation when they were healthy. None of the participants had ever registered for organ donor card in the past when they were healthy. In a study on college students in India, only 2.04% of studied population were registered for organ donation (5).

In a developing country like India where there is a significant shortage of organs for transplantation, there is a strong need to conduct a campaign for increasing the awareness and practice of organ donation. The implications of this study are to emphasize the need to educate people about organ donation and its allocation policies. It can be achieved through the strong mass media campaign, proper implementation of legislation and inculcating in their routine activities regarding its importance. It also requires an organized infrastructure in place for organ procurement, preservation and transplantation, strict guidelines for ethical practice and a strong political will.

## Conclusion

As we expect that affected CKD population will have more knowledge about organ donation as compared to general public, we found that even affected population has less knowledge regarding legislation of organ donation. About 32% of our participants believed that there is a danger that donated organs could be misused, abused or misappropriated. Increasing the awareness about organ donation and allocation procedures can alleviate such problems and misbeliefs. Mass media, religious and political leaders may be involved to maximize awareness about organ donation in general.

## Limitations of study

Limited sample size is the limitation of the study.

## Authors’ contribution

All authors wrote the paper equally.

## Conflicts of interest

The authors declared no competing interests.

## Ethical considerations

Ethical issues (including plagiarism, misconduct, data fabrication, falsification, double publication or submission, redundancy) have been completely observed by the authors.

## Funding/Support

None.

## References

[1] Filho MA, Ramalho H, Pires HS, Silveria JA (1995). Attitudes and awareness regarding organ dona-tion in the western region of Sao Paulo. Transplant Proc.

[2] Rene AA, Viera E, Jiles R, Daniels DE (1995). Organ donation awareness: knowledge, attitude and beliefs in Puerto Rican Population. Transplant Proc.

[3] Odusanya OO, Ladipo CO (2006). Organ donation: knowledge, attitudes, and practice in Lagos, Nigeria. Artif Organs.

[4] Coelho JC, Cilião C, Parolin MB, de Freitas AC, Gama Filho OP, Saad DT (2007). Opinion and knowledge of the population of a Brazilian city about organ donation and transplantation. Rev Assoc Med Bras.

[5] Annadurai K, Mani K, Ramasamy J (2013). A study on knowledge, attitude and practices about organ donation among college students in Chennai, Tamil Nadu. Progress in Health Sciences.

